# Three patients presenting with severe macrosomia and congenital hypertrophic cardiomyopathy: a case series

**DOI:** 10.1186/s13256-017-1231-5

**Published:** 2017-03-24

**Authors:** Marie Vincent, Nadir Benbrik, Bénédicte Romefort, Agnès Colombel, Stéphane Bézieau, Bertrand Isidor

**Affiliations:** 1 Service de Génétique Médicale, Hôpital Hôtel-Dieu, CHU de Nantes, 1 place Alexis Ricordeau, 44093 Nantes, France; 2 Service de Cardiologie pédiatrique, Hôpital Mère-Enfant, CHU de Nantes, 38, boulevard Jean-Monnet, 44093 Nantes, France; 3 Service de Biologie de la Reproduction, Hôpital Mère-Enfant, CHU de Nantes, 38, boulevard Jean-Monnet, 44093 Nantes, France

**Keywords:** Macrosomia, Hypertrophic cardiomyopathy, Genetic, Maternal diabetes, Case report

## Abstract

**Background:**

Macrosomia and hypertrophic cardiomyopathy are two features often associated in neonates of diabetic mothers. We report the cases of three patients with severe macrosomia and critical hypertrophic cardiomyopathy without severely unbalanced maternal diabetes. Only three patients with those two features and no uncontrolled maternal diabetes have been previously reported.

**Case presentation:**

The first patient was a 39-week-old girl, the second patient was a 39-week-old girl, and the third patient was a 41-week-old boy. The two French girls and the French boy had severe macrosomia and hypertrophic cardiomyopathy, leading to the death of the boy. The outcome of the two girls was favorable, with a standardization of growth curves and ventricular hypertrophy. Their mothers presented with high body mass index but no severe documented maternal diabetes; glycemic imbalance was only suspected on postnatal analyses. There was no hydramnios during pregnancy and no other environmental factor, especially toxic exposure. Their parents are from Mayotte, Guadeloupe, and Guinea-Conakry. The usual genetics causes, Beckwith–Wiedemann syndrome, and chromosomal copy number variation, were also excluded.

**Conclusions:**

This report suggests the implication of other factors in addition to glycemic disorders, including genetic factors, in the occurrence of macrosomia and severe hypertrophic cardiomyopathy in neonates. These three original observations indicate that gynecologists and neonatologists should pay attention to neonates from mothers with a high body mass index and when maternal diabetes is not documented.

**Electronic supplementary material:**

The online version of this article (doi:10.1186/s13256-017-1231-5) contains supplementary material, which is available to authorized users.

## Background

Newborn macrosomia is defined as neonate’s birthweight over 4000 g and is usually attributed to maternal obesity, gestational weight gain excess, and diabetes. Macrosomia may also be part of a genetic disorder, such as Beckwith–Wiedemann syndrome (BWS), Online Mendelian Inheritance in Man (OMIM) database number 130650; Simpson–Golabi–Behmel syndrome, OMIM 312870; or Perlman syndrome, OMIM 267000. Cardiomyopathy is not a common feature in those three pathologies.

Hypertrophic cardiomyopathy (HCM) can be sporadic or familial. In familial cases, all modes of inheritance are described, but an autosomal dominant manner is the most common. HCM is also related to gestational diabetes mellitus (GDM), in particular when diabetes is unbalanced in late gestation [1]. HCM was identified as the most common cardiac malformation in fetuses of diabetic mothers, but severe HCM, defined as having an end-diastolic interventricular septal thickness (IVSd) Z-score >2, is rarely described.

There are only a few reports in the literature of patients with macrosomia and severe HCM, for which uncontrolled GDM was not confirmed [2–4]. Here we report on three cases neonates presenting with severe macrosomia and HCM, without extreme maternal diabetes.

## Cases presentation

### Patient 1

The first patient was a 39-week-old baby girl born by cesarean section because of macrosomia. She was the first-born of unrelated and unaffected parents from Mayotte, with no remarkable family history. Pregnancy was uneventful with normal ultrasounds, no hydramnios, and no toxic exposure. Her mother’s height and weight were 176 cm and 115 kg, giving a body mass index (BMI) of 36.5 kg/m2. No oral glucose tolerance test (OGTT) was performed during pregnancy, but 2 days after delivery her mother’s OGTT was in the normal range. Her mother’s postnatal glycated hemoglobin (HbA1C) value was 7.1 % (normal <6 %), which means that her glycemia levels were probably slightly elevated during pregnancy (see details in Additional file 1: Table S1).

**Fig. 1:**
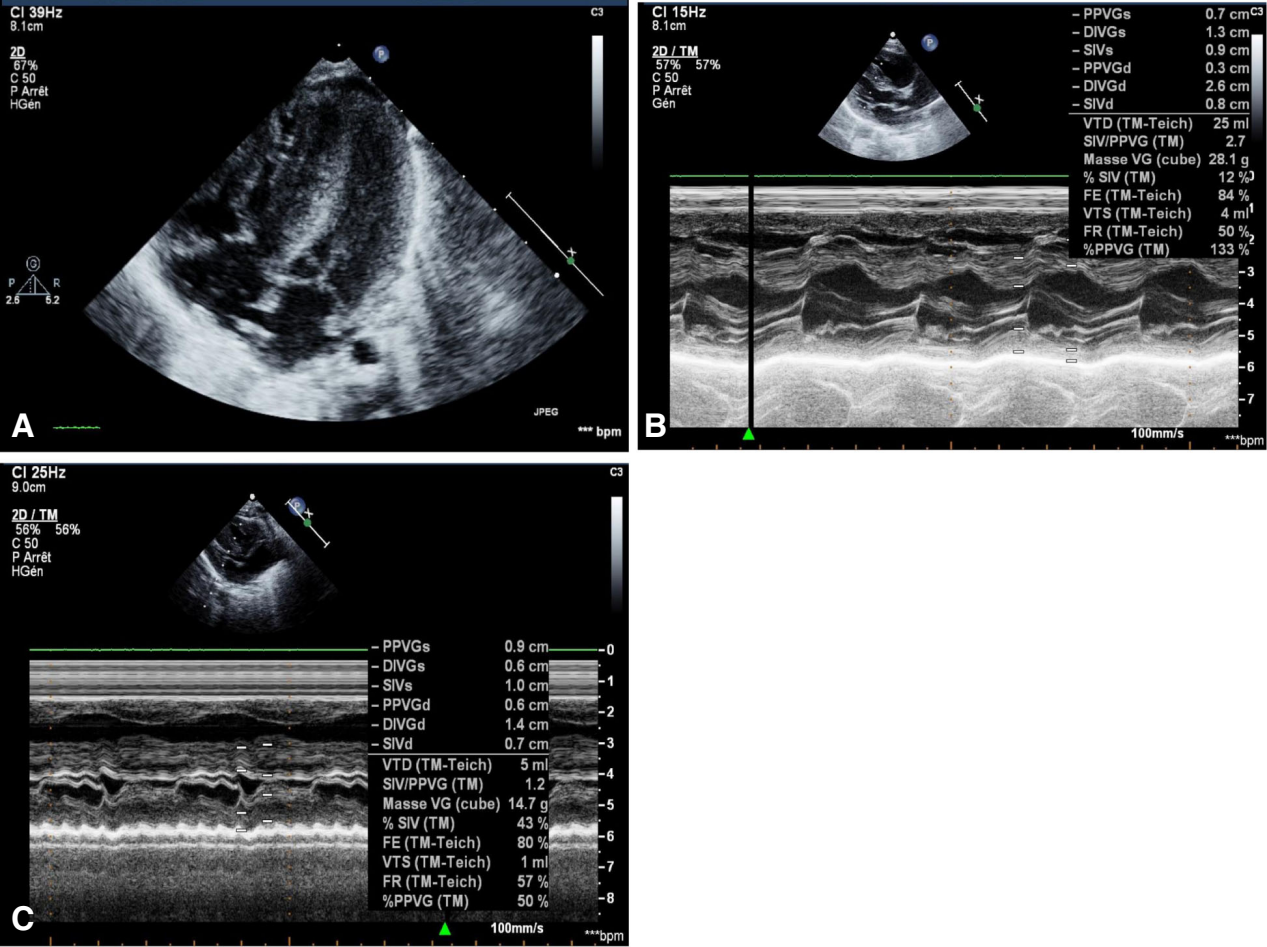
Echocardiography of the three patients. **a** Patient 1. Severe biventricular non-obstructive hypertrophic cardiomyopathy (interventricular septal thickness 12 mm, +5.2 Z-score) and virtual cavity in the *left* ventricle. **b** Patient 2. Hypertrophic cardiomyopathy predominant left (interventricular septal thickness 8 mm, +3.2 Z-score). **c** Patient 3. Severe hypertrophic cardiomyopathy (interventricular septal thickness 7 mm, +2.8 Z-score), with virtual cavity in the *left* ventricle and cardiac function alteration

**Fig. 2:**
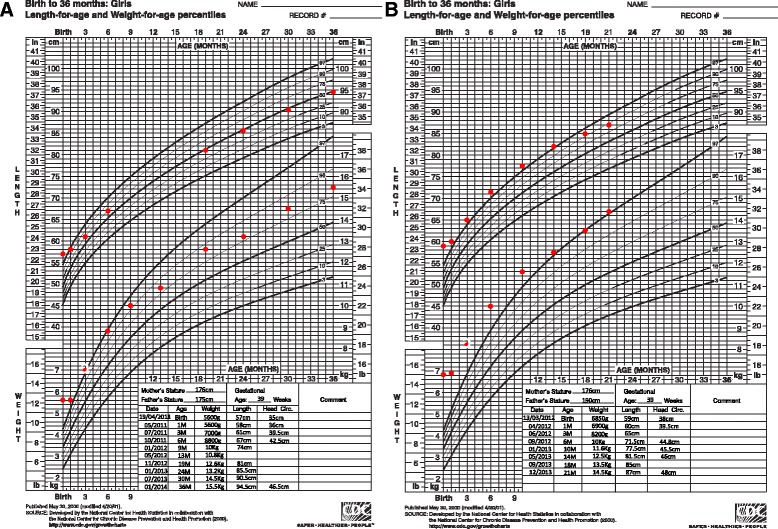
Growth curves of patients 1 and 2. **a** Patient 1. **b** Patient 2. Note the standardization of the measurements

Patient 1’s birth weight (BW) was 5660 g (+4 standard deviation (SD)), birth length (BL) 57 cm (+3 SD), and her birth occipitofrontal circumference (OFC) 37.5 cm (+3 SD). Her Apgar score was 5 at 1 minute and 7 at 5 minutes due to cardiogenic shock with severe pulmonary arterial hypertension. A cardiac ultrasound showed a severe biventricular non-obstructive HCM, with an IVSd at 12 mm (+5.2 Z-score cardiac parameter Dubois) and virtual cavity in her left ventricle (Fig. 1a). Her clinical condition required an extracorporeal membrane oxygenation (ECMO) in emergency for 5 days. She was intubated for 11 days, with 2 days of high frequency oscillation (HFO). Metabolic investigations and cerebral magnetic resonance imaging (MRI) were normal. HCM decreased in 2 weeks and macrosomia disappeared (Fig. 2a). At 36 months, a cardiac ultrasound showed a residual hypertrophy of her left ventricle at 3 mm, with an aspect of thick and hyperechogenic myocardium. Her weight was 15.5 kg (+1.5 SD), her length was 94.5 cm (+0.5 SD), and her OFC was 46.5 cm (–1.5 SD; Fig. 2a). Her psychomotor development was normal.

Patient 1’s mother developed documented slight GDM during her following pregnancy, which was only treated with diet; her newborn did not have macrosomia or HCM.

### Patient 2

The second patient was a 39-week-old baby girl born by cesarean section because of fetal arrhythmia and macrosomia. She was the fourth-born of unrelated and unaffected parents from Guadeloupe. Her family history was unremarkable. Her mother’s and father’s weight and height were 80 kg and 176 cm (BMI 25.5 kg/m2), and 130 kg and 190 cm. The BWs of her mother, father, and her siblings were respectively 3000 g, 4000 g, 4400 g, 4900 g and 4700 g. Pregnancy was uneventful with normal ultrasounds until 38 weeks; there was no hydramnios and no toxic exposure. Her mother’s OGTT at 22 weeks was normal and HbA1C was also in the normal range at 5.5 % 10 days after delivery (see details in Additional file 1: Table S1).

The BW of Patient 2 was 6850 g (+6.5 SD), BL was 59 cm (+4 SD), and birth OFC was 38 cm (+3 SD). She required immediately oxygen for transient neonatal respiratory distress; a cardiac ultrasound revealed HCM (IVSd 8 mm, +3.2 Z-score cardiac parameter Dubois; Fig. 1b). Molecular analysis of BWS was negative, and deoxyribonucleic acid (DNA)-microarray was normal.

At 21 months, her weight was 14.5 kg (+3 SD), her length was 87 cm (+2 SD), and her OFC was 48 cm (+1 SD; Fig. 2b). Her psychomotor development was normal and a cardiac ultrasound showed mild HCM.

### Patient 3

The third patient was a 41-week-old baby boy born by cesarean section because of fetal arrhythmia and decreased fetal movements. He was the first-born of unrelated and unaffected parents from Guinea-Conakry. His family history was uneventful. His mother’s height and weight were 173 cm and 80 kg, giving a BMI of 27 kg/m2. Antenatal ultrasounds were normal, without hydramnios, and no drug or toxic exposition was documented. There was no suspicion of maternal diabetes, so OGTT was not performed during pregnancy; his mother’s OGTT was normal 3 days after delivery (see details in Additional file 1: Table S1).

His BW was 5000 g (+5.5 SD), BL 54 cm (+3 SD), and birth OFC 37 cm (+2 SD). He was born in a state of apparent death and was directly intubated for artificial ventilation with HFO because of refractory hypoxemia. He developed pulmonary hypertension at day 5 and echocardiography revealed severe HCM (IVSd 7 mm, +2.8 Z-score cardiac parameters Dubois; Fig. 1c). Several episodes of hypoglycemia were documented and a cerebral MRI revealed massive ischemic and hemorrhagic lesions. Death occurred at the fifth day of life. An autopsy was not done because of parental refusal. BWS molecular analysis was normal.

## Discussion

HCM is a well-known complication in babies of diabetic mothers [5–8], and is attributed to a compensatory increase in fetal insulin secretion. While HCM in babies of diabetic mothers is usually mild and reversible, it is rarely severe leading to fetal or neonatal death. Only five patients with severe neonatal HCM were reported previously in association with GDM [2, 3, 9–11]. Other studies showed that glucose and insulin levels were not so predictive of fetal macrosomia [12, 13].

Sardesai *et al*. reported the case of a fetus with fatal HCM and macrosomia. The mother had a well-controlled GDM under insulin therapy [2]. Moreover, Mehta and Hussain described another patient with transient hyperinsulinism associated with macrosomia, severe obstructive HCM, hepatomegaly, and nephromegaly in a mother with mild GDM requiring no treatment [3]. Maternal postnatal OGTT was negative, and follow-up showed an improvement of the HCM with a mild residual left ventricular hypertrophy. In those two last case reports, it seems difficult to attribute the severe features observed only to a benign GDM.

Furthermore, Godfried and coworkers reported the case of a patient with severe HCM, macrosomia (BW 5290 g), and hepatomegaly born to a mother with human immunodeficiency virus (HIV) infection [4]. GDM and other metabolic disorders were excluded, and a relationship between the neonatal abnormalities and antiretroviral treatment was suspected. Other toxic causes have not been reported in the literature and there was no history of toxic exposure for our three patients.

Maternal diabetes was excluded in the mother of Patient 2. OGTT of the mothers of Patients 1 and 3 were not performed during pregnancy but postnatal analyses do not indicate severe glycemia disequilibrium. Even if we consider mild GDM in those two cases, this cannot explain completely, in our opinion, the severity of the children’s phenotype.

The three major genetic overgrowth syndromes including BWS, Simpson–Golabi–Behmel syndrome, and Perlman syndrome, do not usually present with cardiomyopathy. Cardiomyopathy has been rarely reported in BWS [14, 15], but molecular analysis ruled out this hypothesis in Patients 2 and 3.

To date, more than 13 genes have been identified for isolated HCM. Most of them are inherited in an autosomal dominant pattern. In the present report we did not consider monogenic forms of HCM because of macrosomia, the spontaneously favorable evolution, and the absence of family history.

## Conclusions

We reported the case of three patients with macrosomia and severe HCM at birth. We hypothesize that other additional factors to maternal diabetes, such as genetic and/or metabolic factors, might be involved in the determinism of this phenotype. We propose that gynecologists should perform OGTT during pregnancy in cases of high BMI, even if there is no familial history of GDM or hydramnios. We propose also that neonatologists and pediatric cardiologists pay special attention to neonates presenting with macrosomia, even if no GDM has been documented, and pay attention to other potential factors.

## Additional file

Additional file 1: Table S1. Mothers’ oral glucose tolerance test (OGTT) of patients 1, 2, and 3. (XLS 20 kb)

## Abbreviations

BL: Birth length
BMI: Body mass index
BW: Birth weight
BWS: Beckwith–Wiedemann syndrome
ECMO: Extracorporeal membrane oxygenation
GDM: Gestational diabetes mellitus
HbA1C: Glycated hemoglobin
HCM: Hypertrophic cardiomyopathy
HFO: High frequency oscillation
IVSd: Interventricular septal thickness
OFC: Occipitofrontal circumference
OGTT: Oral glucose tolerance test
OMIM: Online Mendelian Inheritance in Man
